# Ten years to VISION 2020: how are we doing?

**Published:** 2010-12

**Authors:** Peter Ackland

**Affiliations:** Chief Executive, International Agency for the Prevention of Blindness (IAPB), London School of Hygiene and Tropical Medicine, Keppel Street, London WC1E 7HT, UK.

**Figure:**
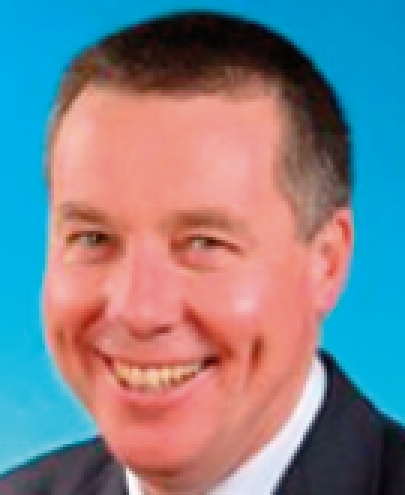


## An encouraging start

We have just passed the halfway mark for the VISION 2020 global initiative, which was launched in 1999 with the goal to eliminate avoidable blindness by the year 2020. This is a good time to take stock of what we have achieved and what still needs to be done.

The success of VISION 2020 has to be judged against its impact on reducing levels of avoidable blindness in the world. Although still to be finally approved by WHO, some preliminary data on the global prevalence of blindness and visual impairment was presented in a meeting between WHO and IAPB members in October 2010. It suggests a decline of approximately 10% in the overall number of blind and visually impaired. Compared to the 314 million people with visual impairment (≤6/18) from WHO data produced in 2004, the new figures suggest a total of 285 million. Overall, this is a decrease of nearly 29 million. The number of blind people (≤3/60, presenting vision) has fallen from an estimated 45 million to 39.8 million. If these figures are confirmed, and if we take into account that, over the same period, there has been an 18% increase in the population of those aged 50 years and older worldwide, then we have some cause for optimism.

We also know that:

- The prevalence of blindness is decreasing in some countries that have adopted VISION 2020 strategies. The most recent national studies done in Pakistan, India, and The Gambia have all shown significant declines in prevalence rates compared to earlier surveys.
- The number of cataract operations done in India has increased fivefold over the past 25 years, to more than 5 million per year, and the lessons learnt are having a major positive impact in other countries.
- Blindness due to trachoma and onchocerciasis has decreased significantly and the possibility of the elimination of transmission of these two diseases by the year 2020 is within reach.
- Childhood blindness is decreasing due to vitamin A supplementation, measles immunisation, and the focus on blinding conditions such as retinopathy of prematurity.
- Half of the world's visual impairment is due to uncorrected refractive error, and significant progress has been made in bringing refraction and spectacle making to the poorest communities.

## Scaling up and adopting new strategies

But much more needs to be done if we are to achieve our overall objective. The way forward will require us to build upon existing success, to ‘scale up’ what we are already doing (by going from project level to full country-wide programmes), and to adopt new strategies where progress has been slower than hoped.

For VISION 2020, increasing the available financial resources to implement national VISION 2020 plans and to bring good quality, equitable eye health services to the poorest communities is one very obvious area that requires our focus going forward. This will require extensive advocacy work, itself based on sound evidence, to influence and change the minds of policy makers around the world, most of whom presently see blindness as a low priority. More advocacy and more targeted research to prove our case (see article on page 43) are vital to our future progress.

But even if we were able to get more money, would countries have the capacity to absorb it and actually deliver the much-needed eye health services? Sadly, the answer is no in many countries - because of the chronic shortage of eye health workers. Human resource development for eye health must receive even greater emphasis in the second decade of VISION 2020. Training is an important aspect of this but only one part of a complex jigsaw that includes wider policy issues such as staff retention and motivation, deployment to rural areas, the ‘brain drain’ to high-income countries and/or private practice, and so on.

Another important area to consider is the creation of consumer demand for eye health services. Why do so many people still turn to traditional treatments rather than seek out the eye units that VISION 2020 has so busily promoted? There are many reasons and this is not the place to investigate them in detail. But quality and access have to receive even greater attention than previously. For example, the quality of outcomes for cataract and trichiasis surgery is unacceptable in many countries and standards of surgery have to be improved.

**‘We have to look for opportunities to promote VISION 2020 within the wider health development world’**

**Figure:**
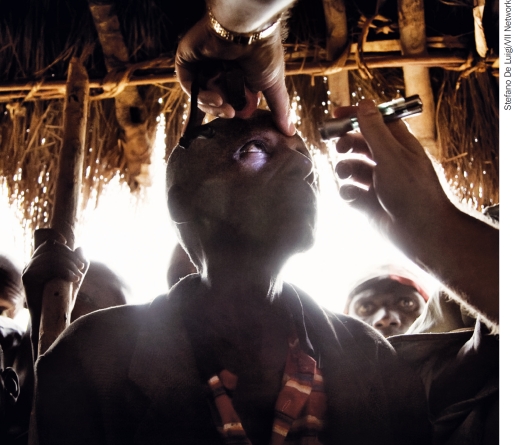
Checking a patient for cataract. BURUNDI

Stefano De Luigi/VII Network

We also have to look for opportunities to promote VISION 2020 within the wider health development world. For example:

- The current emphasis of many of the big donor agencies is to support the strengthening of health systems, rather than fund individual vertical initiatives. At the very least, we shall need to consider how current VISION 2020 approaches align with broader health system development.
- The global shortage of health workers is a very serious problem that extends far beyond eye care -we cannot resolve our own need for more eye health personnel without taking account of initiatives such as the Global Health Workforce Alliance.
- There are opportunities for us to engage with the reawakened global interest in primary health care.

All of the above will require us to make new partnerships that take us outside our traditional comfort zone within our own profession.

This may all seem rather daunting, but we must remember that there has been a huge amount of innovation and progress within VISION 2020. We have much to contribute to the world of health development and others can learn as much from us as we can from them.

**Scaling up**

‘Scaling up’ is a commonly used term in development circles - but what does it mean? Recently, interesting work has been done to think through what scaling up really means in terms of international health. One approach is to consider the barriers that are currently preventing health approaches from being taken to scale. Take a look at **www.expandnet.net** for more information on this interesting topic.

